# Long-Term Culture of Astrocytes Attenuates the Readily Releasable Pool of Synaptic Vesicles

**DOI:** 10.1371/journal.pone.0048034

**Published:** 2012-10-26

**Authors:** Hiroyuki Kawano, Shutaro Katsurabayashi, Yasuhiro Kakazu, Yuta Yamashita, Natsuko Kubo, Masafumi Kubo, Hideto Okuda, Kotaro Takasaki, Kaori Kubota, Kenichi Mishima, Michihiro Fujiwara, N. Charles Harata, Katsunori Iwasaki

**Affiliations:** 1 Department of Neuropharmacology, Faculty of Pharmaceutical Sciences, Fukuoka University, Fukuoka, Fukuoka, Japan; 2 A.I.G. Collaborative Research Institute for Aging and Brain Sciences, Fukuoka University, Fukuoka, Fukuoka, Japan; 3 Department of Molecular Physiology and Biophysics, University of Iowa Carver College of Medicine, Iowa City, Iowa, United States of America; Virginia Tech Carilion Research Institute, United States of America

## Abstract

The astrocyte is a major glial cell type of the brain, and plays key roles in the formation, maturation, stabilization and elimination of synapses. Thus, changes in astrocyte condition and age can influence information processing at synapses. However, whether and how aging astrocytes affect synaptic function and maturation have not yet been thoroughly investigated. Here, we show the effects of prolonged culture on the ability of astrocytes to induce synapse formation and to modify synaptic transmission, using cultured autaptic neurons. By 9 weeks in culture, astrocytes derived from the mouse cerebral cortex demonstrated increases in β-galactosidase activity and glial fibrillary acidic protein (GFAP) expression, both of which are characteristic of aging and glial activation *in vitro*. Autaptic hippocampal neurons plated on these aging astrocytes showed a smaller amount of evoked release of the excitatory neurotransmitter glutamate, and a lower frequency of miniature release of glutamate, both of which were attributable to a reduction in the pool of readily releasable synaptic vesicles. Other features of synaptogenesis and synaptic transmission were retained, for example the ability to induce structural synapses, the presynaptic release probability, the fraction of functional presynaptic nerve terminals, and the ability to recruit functional AMPA and NMDA glutamate receptors to synapses. Thus the presence of aging astrocytes affects the efficiency of synaptic transmission. Given that the pool of readily releasable vesicles is also small at immature synapses, our results are consistent with astrocytic aging leading to retarded synapse maturation.

## Introduction

Astrocytes play a critical role in regulating information processing at synapses of the central nervous system [1], [2]. Moreover, in their presence, the number of synapses increases dramatically [3], [4], [5], suggesting that astrocytes provide neurons with the energy and secreted substrates they need for the formation, maturation and stabilization of functional synapses [1], [2]. Previous reports have demonstrated that synaptic maturation is strongly influenced by chemical factors that are secreted by astrocytes. Examples include: cholesterol complexed to apolipoprotein E-containing lipoproteins [6]; the extracellular matrix proteins thrombospondins [7]; the matricellular proteins hevin and secreted protein acidic and rich in cysteine (SPARC) [8], [9]; and glypicans 4 and 6 [10]. In addition to secreting these factors, astrocytes can regulate synaptogenesis through intricate physical interactions with neurons [11]. For instance, physical contact with neurons via neural integrin receptors activates protein kinase C within neurons; this diffusible intracellular messenger contributes to synaptogenesis [5]. Thus, synaptic maturation and neuronal activity are both under many forms of astrocytic control.

The above-mentioned studies were carried out using young astrocytes. Notably, a recent study reported age-dependent changes in the electrical responses in astrocytes when neurons were stimulated *in situ* [12]. Whether the presence of aged astrocytes also impacts neuronal function is unknown. Based on the roles of young astrocytes, we speculate that the aged astrocytes can also influence synaptic transmission and synaptogenesis. However, an *in vivo* study of the aging brain would be complicated by the fact that not only astrocytes, but also the neurons, will age; thus any observed changes in neuronal function could be consequences of change in the astrocytes, the neurons, or both. Study of the impacts of aged astrocytes would be further complicated by the presence of proliferating (newly generated) astrocytes that are present even in adult brains [13], [14], [15], [16]. Thus, the effects of astrocytic senescence have been difficult to distinguish from those of neuronal senescence and of astrogenesis.

In this study, we evaluated how astrocytes of different ages influence synaptic transmission in the absence of astrogenesis. We utilized long-term cultures of cerebral cortical astrocytes, as this enabled us to control the ages of neurons and astrocytes separately. Specifically, we plated neurons on a feeder layer comprising astrocytes of a particular age (5, 9 or 16 weeks old), and evaluated differences in synaptic transmission among the test groups. Antimitotic reagents were used to suppress the effects of astrogenesis. Thus our study takes advantage of a cell culture system that has become useful for understanding mechanisms that underlie the development, maturation and senescence of neurons and astrocytes [17], [18], [19], [20], [21], [22], [23], [24]. Here we report that excitatory synaptic transmission is attenuated in the presence of aged astrocytes, owing to a reduction in the readily releasable pool (RRP) of synaptic vesicles, in the absence of changes in other synaptic functions or synapse formation.

## Results

### *In vitro* Model of Senescent Astrocytes

**Figure 1:**
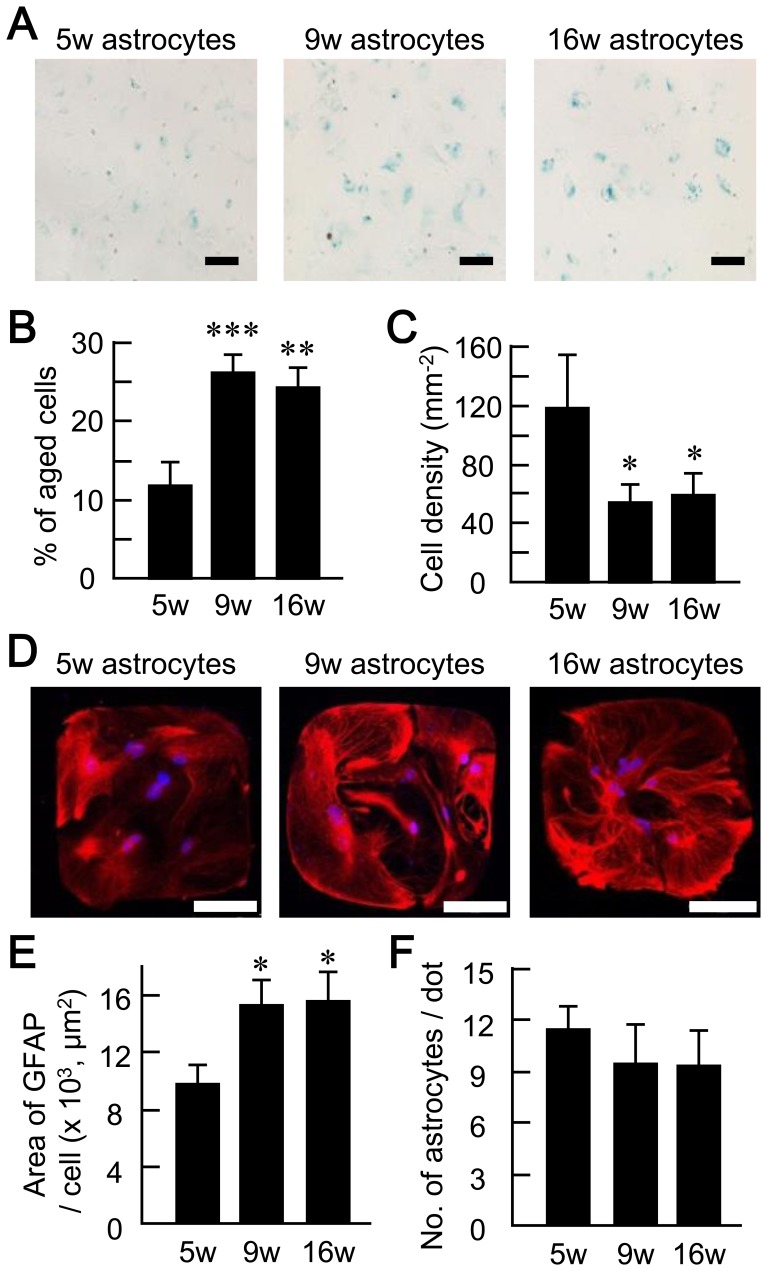
Astrocytes cultured for long periods exhibit detectable aging at the 9- and 16-week time points. (A) Representative bright-field images of astrocytes mass-cultured for 5, 9, and 16 weeks *in vitro*. In each case, cells were positive for β-galactosidase activity (blue), but the frequency of such cells was higher in cells cultured *in vitro* for a prolonged period. Scale bars, 100 µm. (B) Percentages of astrocytes positive for β-galactosidase activity in mass cultures 5-, 9- and 16-weeks of age. (C) Density of astrocytes based on nuclear counterstaining with DAPI, in mass cultures 5-, 9- and 16-weeks of age (*N* = 3 cultures at each age, with each culture representing 3 image fields). Data were obtained from the same culture as in (B). (D) Representative immunostaining of astrocyte microislands cultured *in vitro* for 5, 9, and 16 weeks. The cells were immunostained for the astrocyte marker GFAP (red), and counterstained with DAPI to detect nuclei (blue). Note that the area positive for GFAP immunoreactivity increased with astrocyte age. Scale bars, 100 µm. (E) The area per astrocyte that was positive for GFAP staining. This value is calculated by dividing the measured area by the number of astrocytes in each microisland. (F) The number of astrocytes in each microisland, following culture for 5, 9 and 16 weeks (*n* = 30 microislands, respectively). Data were obtained from the same culture as in (E). *, p<0.05; **, p<0.01; ***, p<0.001.

We first assessed whether cerebral cortical astrocytes maintained in long-term culture show morphological features characteristic of senescence (Figure 1). Astrocytes in mass culture (free of neurons) were assessed for endogenous β-galactosidase activity at pH 6. Such activity distinguishes between senescent versus quiescent, immortal, or tumor cells [17], [41] and is used alongside other indicators (e.g. as the generation of reactive oxygen species, reduced mitochondrial activity, and increased expression of haeme oxygenase-1) to establish to what extent cultured cells *in vitro* have aged [17], [18], [42]. By 5 weeks (5w) in culture, some astrocytes were positive for β-galactosidase activity and the number increased in cells cultured for longer periods (Figure 1A), as reported previously for astrocytes mass cultured for 90 days *in vitro* [17]. The percentage was significantly higher in both 9- and 16-week-old cultures than in 5-week-old cultures (5 weeks *in vitro* (w), 11.5±2.8%; 9w, 26.1±2.1%; 16w, 24.0±2.3%, Figure 1B). Thus the ß-galactosidase activity started to increase before 9 weeks of culture and was stable at that level for up to 16 weeks. The increase in ß-galactosidase staining was accompanied by a decrease in astrocyte density (Figure 1C), as demonstrated by nuclear counterstaining with DAPI in the same culture. The densities of the astrocytes in 9- and 16-week-old cultures were reduced to half of that in the 5-week-old cultures (5w, 117.13±35.4/mm^2^; 9w, 52.67±11.7/mm^2^; 16w, 58.22±13.3/mm^2^).

We next prepared neuron-free microislands of astrocytes to use for neuronal plating and electrophysiological studies. The levels of astrocytic proteins, such as glial fibrillary acidic protein (GFAP) and S100ß, have been reported to increase in astrocytes that have undergone more than 40 DIV [17], [43], [44], [45]. We evaluated this phenomenon by immunohistochemical staining for GFAP (Figure 1D), and measurement of the area of GFAP staining per astrocyte in microisland cultures (Figure 1E). For this purpose, the area was measured as the number of pixels whose intensity was above a certain threshold and therefore it corresponds to the amount of expression. The GFAP levels were significantly increased in 9- and 16-week-old vs. 5-week-old cultures (5w, 9664.8±1245.9 µm^2^/cell; 9w, 15151.0±1753.0 µm^2^/cell; 16w, 15409.0±2020.6 µm^2^/cell). Similarly as in ß-galactosidase activity (Figure 1B), GFAP expression started to increase before 9 weeks of culture and was stabilized up to 16 weeks.

In the case of microisland cultures, the astrocytes were plated at modified densities, so that the densities after 5, 9 and 16 weeks *in vitro* were the same by factoring in the decreases in astrocyte density during long-term culture (Figure 1C). Nuclear counterstaining with DAPI showed that the numbers of astrocytes per microisland (dot) were indistinguishable among the three groups (5w, 11.4±1.2; 9w, 9.4±2.2; 16w, 9.3±2.0, Figure 1F). The β-galactosidase staining in microisland cultures showed only a few number of positive astrocytes (data not shown) and was not analyzed, because the β-galactosidase staining was positive in low percentage of cells (Figure 1B), and that the number of astrocytes in microisland culture was low (Figure 1F).

The demonstration that 9- and 16-week-old cultures exhibited parallel increases in ß-galactosidase activity (Figure 1B) and GFAP expression (Figure 1E) was consistent with previous reports on senescence. We therefore consider the astrocytes cultured for 5 weeks as pre-senescence controls, and those cultured for 9 weeks (∼63 DIV) or longer as an *in vitro* model of astrocytic senescence.

### Long-term Culture of Astrocytes Suppresses Evoked Synaptic Transmission

To evaluate the impacts of senescent astrocytes on synaptic transmission, we used an autaptic neuron culture preparation [25], [26], [34], [35], [36], [37], [38], [39]. Single neurons plated on astrocyte microislands make synapses onto their own dendrites and somata (autapses), in areas that are bounded by the astrocytic microisland edges. This preparation enables the recording of electrical responses from all synapses (autapses) of a neuron, which is not possible in mass culture. It also allows for a more quantitative analyses of electrophysiological responses in that it provides better control of membrane voltage than in neurons in mass cultures.

Hippocampal neurons obtained from newborn mice were plated on microisland astrocytes that had been cultured *in vitro* for 3, 7 or 14 weeks (see Methods). Following synaptic experiments were performed between 13 and 16 DIV of neuronal age, i.e., the astrocytes were cultured for a total of 5, 9 and 16 weeks, as in the above experiments on morphological features (Figure 1), by the time the features of neuronal synapses were analyzed. The synaptic features in older cultures of astrocytes (9 and 16 weeks of astrocytic age) were compared to those in control sister cultures (5 weeks of astrocytic age) in the following studies.

**Figure 2:**
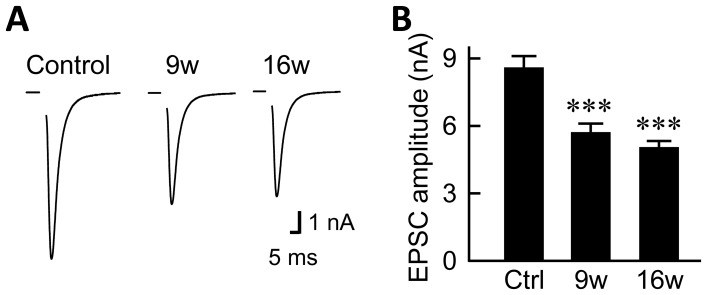
Astrocyte aging suppresses evoked synaptic transmission. (A) Representative traces of evoked EPSCs recorded from autaptic hippocampal neurons co-cultured with astrocytes that had been cultured for 5, 9 or 16 weeks (control, 9w and 16w) by the time of electrophysiological recording. The holding potential (Vh) was step-depolarized from −70 to 0 mV for 2 ms, and returned to −70 mV. Depolarization artifacts caused by the generated action currents have been removed for clarity. (B) Average amplitudes of the evoked EPSCs in neurons co-cultured with 5- (control), 9- and 16-week-old astrocytes (*n* = 80, 82 and 86 autaptic neurons, respectively). ***, p<0.001.

As a lumped synaptic event involving both pre- and postsynaptic components, we examined the excitatory postsynaptic current (EPSC) evoked by an action potential (Figure 2). Step depolarization of the patch-clamped neuron using a patch pipette generated an action potential and induced the release of neurotransmitters from synaptic vesicles. The recorded EPSC was blocked by 10 µM CNQX, a competitive antagonist of AMPA-subtype of the ionotropic glutamate receptors (data not shown). This result suggests that the recorded synaptic responses were mediated by presynaptic release of the excitatory neurotransmitter glutamate, followed by postsynaptic activation of AMPA receptors, as reported previously [25].

In cultures of 9-week-old astrocytes, the amplitude of the evoked EPSC was decreased relative to that in control, 5-week-old astrocytes (Figure 2A). A similar reduction was observed in the cultures of 16-week-old astrocytes. The suppressive effect of older astrocyte cultures was statistically significant (control, 8.52±0.49 nA; 9w, 5.65±0.34 nA; 16w, 4.95±0.28 nA, Figure 2B). The decreased amplitude of the evoked EPSC could have resulted either from reduced EPSCs at individual synapses in response to action potentials, or from the failure to generate an action potential that invades all nerve terminals. In order to exclude the latter possibility, we analyzed the amplitudes of the voltage-dependent Na^+^ and K^+^ currents that underlie the action potentials (Figure S1A) [46]. The amplitude of neither the Na^+^ current (control, 14.44±0.51 nA; 9w, 14.24±0.57 nA; 16w, 14.37±0.56 nA, Figure S1B) nor the K^+^ current (control, 4.17±0.27 nA; 9w, 4.03±0.26 nA; 16w, 4.17±0.29 nA, Figure S1C) changed with the length of the culture period. These data suggest that the decreased amplitude of the evoked EPSC in the context of astrocytic senescence is not due to a failure to generate or conduct an action potential, but rather to purely synaptic causes after an action potential reaches a nerve terminal.

### Long-term Culture of Astrocytes has Presynaptic Effects on Miniature Synaptic Transmission

**Figure 3:**
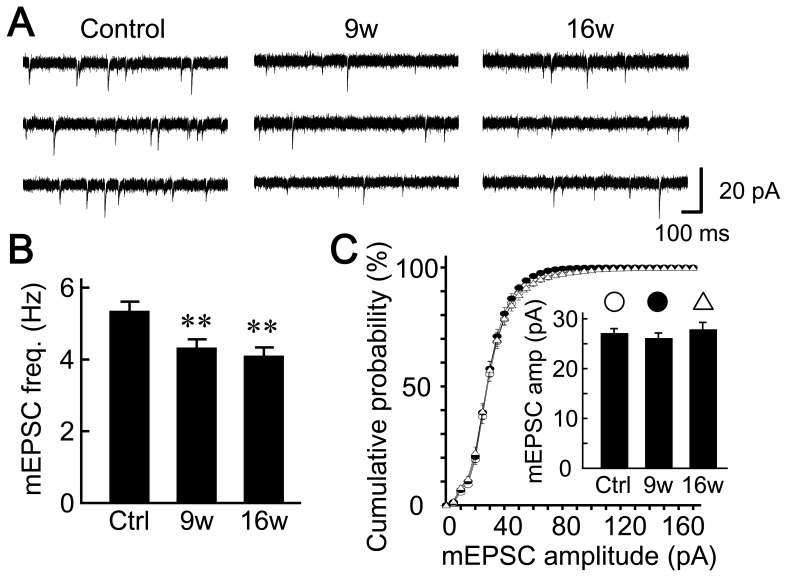
Astrocyte aging reduces presynaptic mechanisms of synaptic transmission. (A) Representative traces of mEPSCs recorded in the presence of 0.5 µM TTX. The Vh was −70 mV. (B) Frequencies of mEPSC events observed in neurons co-cultured with 5- (control), 9- and 16-week-old astrocytes (*n* = 64, 58 and 58 autaptic neurons, respectively). (C) Amplitudes of mEPSCs. The cumulative histogram shows the amplitudes measured from 19,990 (control), 14,806 (9-week) and 13,924 mEPSC events (16-week old astrocytes), with a bin size of 5 pA. *Inset* shows the amplitudes of mEPSCs (means of mean amplitudes) recorded from individual neurons. Data were obtained under the same experimental conditions as in (B). **, p<0.01.

The synaptic causes of the decrease in amplitude of evoked EPSCs can be presynaptic or postsynaptic in origin. In order to evaluate the contribution of each, the miniature EPSCs (mEPSCs) were recorded in the presence of 0.5 µM TTX and in the absence of stimulation (Figure 3). These miniature events arise from the activation of postsynaptic neurotransmitter receptors by single “packets” of neurotransmitter, i.e., the amount released by a single synaptic vesicle [36], [47] independently of action potentials (Figure 3A). As in the case of evoked EPSCs, the recorded mEPSCs were blocked by 10 µM CNQX (data not shown), suggesting that these events are mediated by glutamate-triggered AMPA-receptor activation.

The measured frequency of mEPSCs was decreased in astrocytes cultured for long periods (control, 5.28±0.30 Hz; 9w, 4.25±0.27 Hz; 16w, 4.02±0.27 Hz, Figure 3B). This result implies that synaptic changes have a presynaptic origin. In contrast, the amplitude of the mEPSCs did not change (control, 26.93±1.00 pA; 9w, 25.94±1.02 pA; 16w, 27.66±1.43 pA, Figure 3C), suggesting that there was also no change in the density or sensitivity of postsynaptic AMPA receptors, or in the glutamate content in the vesicles of presynaptic nerve terminals.

To confirm the lack of postsynaptic changes, we bypassed the presynaptic mechanisms and recorded the responses to AMPA-receptor activation (Figure S2). AMPA receptors were directly activated when 10 µM glutamate was applied to the extracellular solution in the presence of the NMDA receptor antagonist APV (50 µM, Figure S2A). As expected, the amplitude of the AMPA receptor-mediated current was unchanged (control, 0.34±0.05 nA; 9w, 0.35±0.04 nA; 16w, 0.37±0.04 nA, Figure S2B). This result supports the notion that the postsynaptic AMPA receptors remain unchanged in the context of astrocytes cultured long-term, and thus the idea that aged astrocytes would influence synaptic transmission via presynaptic mechanisms.

### Long-term Culture of Astrocytes Reduces the Size of the Readily Releasable Pool of Synaptic Vesicles

A reduction in glutamate release in the presence of aging astrocytes could be attributable to a decrease in: 1) RRP size, i.e., the number of synaptic vesicles released from the readily releasable pool; 2) the vesicular release probability (P_vr_), i.e., the probability that a single vesicle will be released from the RRP in response to an action potential; 3) the paired-pulse ratio (PPR) of responses, an indirect readout of probability of release that is a broader concept than P_vr_; or 4) the number of nerve terminals that make synapses on the recorded neuron.

**Figure 4:**
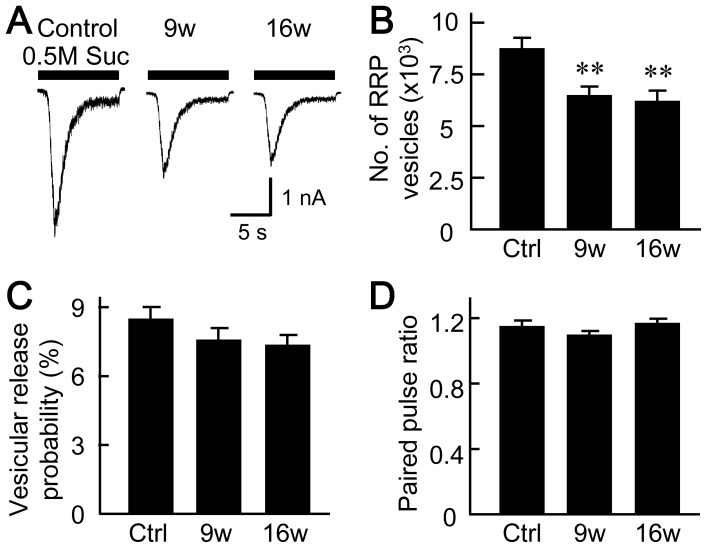
Astrocyte aging significantly reduces the size of the readily releasable pool of synaptic vesicles. (A) Representative traces of the responses to 0.5 M sucrose (10 s) in autaptic neurons co-cultured with 5- (control), 9- and 16-week-old astrocytes. The response to the hypertonic sucrose solution was used to define neurotransmitter release from all vesicles in the readily releasable pool (RRP). The Vh was −70 mV. (B) Number of synaptic vesicles in RRP of autaptic neurons co-cultured with 5- (control), 9- and 16-week-old astrocytes (*n* = 64, 58 and 58 neurons, respectively). (C) Vesicular release probability (P_vr_) in single autaptic neurons co-cultured with 5- (control), 9- and 16-week-old astrocytes (*n* = 80, 82 and 82 neurons, respectively). (D) Paired-pulse ratio (PPR) of evoked EPSCs. The amplitudes of EPSCs evoked by two action potentials separated by 50 ms were measured. Autaptic neurons were co-cultured with astrocytes cultured for 5- (control), 9- and 16-week (*n* = 64, 58 and 58 neurons, respectively). **, p<0.01.

RRP size was assessed based on the ability of hypertonic sucrose solution to force all vesicles in the RRP to release glutamate (Figure 4A) [25], [40]. The number of synaptic vesicles in the RRP was measured by dividing the total electric charge corresponding to the RRP by the mean electric charge of a single vesicle in the same neuron (see Methods for details). RRP size was lower in autaptic neurons cultured with aged astrocytes than in controls (control, 8646±592 vesicles; 9w, 6369±452 vesicles; 16w, 6133±509 vesicles, Figure 4 B).

The P_vr_ was assessed based on the fact that an action potential evokes a release from a small fraction of RRP, and that the same RRP is shared between the glutamate releases evoked by action potentials and hypertonic sucrose solution (Figure 4C) [40]. It was measured by dividing the electrical charge of an EPSC evoked by an action potential by the total electric charge of RRP (see Methods for details) [25], [26]. No statistically significant differences in P_vr_ were detected among the groups tested (control, 8.43±0.49%; 9w, 7.48±0.51%; 16w, 7.25±0.45%).

The paired-pulse ratio (PPR) is defined as the ratio of the amplitude of the second EPSC to that of the first EPSC, when the two EPSCs were evoked by action potentials at short intervals (e.g. 50 msec, Figure 4D). This parameter is an indirect readout of the release probability of a nerve terminal. Specifically this is composed of the P_vr_, the number of vesicles released at a terminal per action potential, the degree to which the glutamate receptor is saturated by a single quantum of glutamate, as well as the sensitivity of the vesicle release machinery to Ca^2+^, whose concentration increases with action potential and which triggers vesicle release [48]. The PPR did not differ significantly among the groups that were tested (control, 1.14±0.03; 9w, 1.09±0.02; 16w, 1.16±0.03, Figure 4D). The lack of change in either P_vr_ or PPR also strongly implied that the sensitivity of the vesicle-release machinery to Ca^2+^ is unchanged in long-term culture of astrocytes.

### Long-term Culture of Astrocytes Affects Dendrite Branching

**Figure 5:**
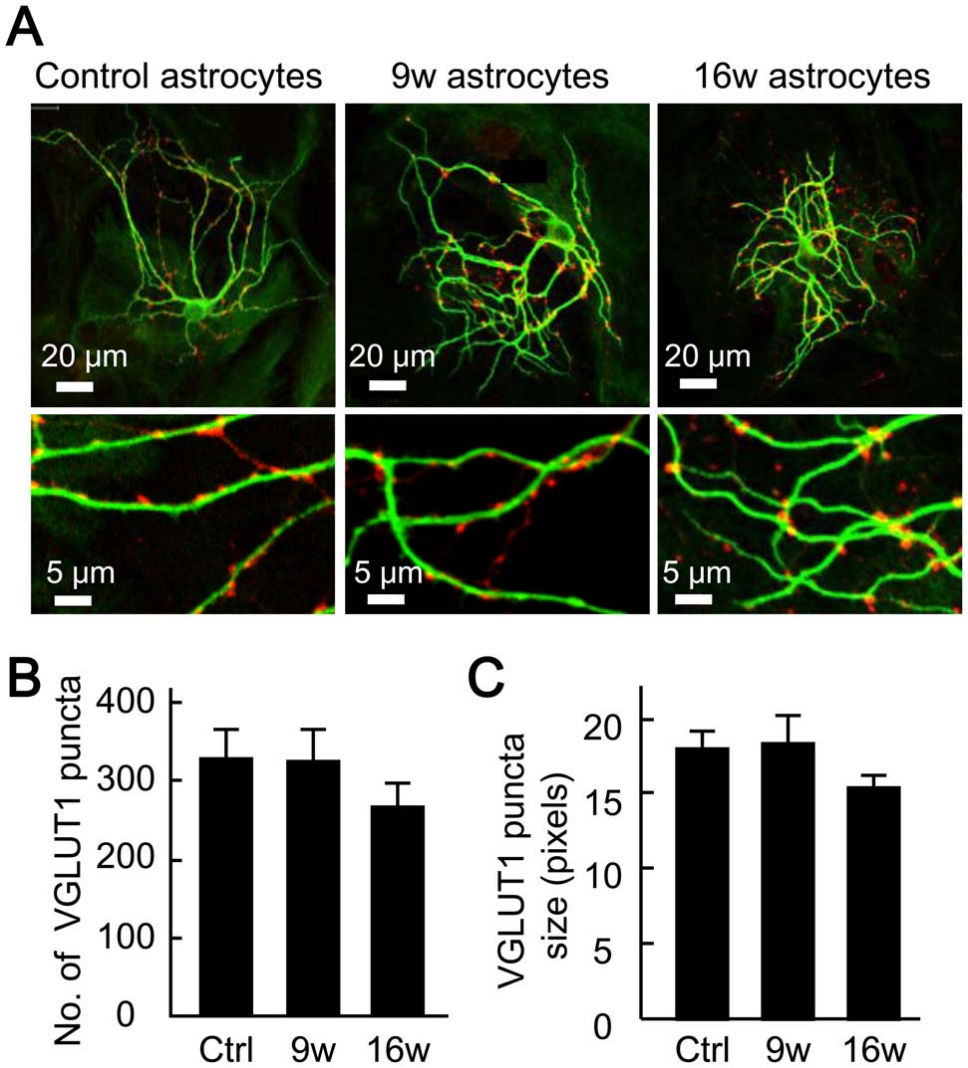
Astrocyte aging does not affect the number of synapses. (A) Representative images of autaptic neurons immunostained for the dendritic marker MAP 2 (green) and the excitatory nerve terminal marker VGLUT 1 (red). Parts of images in the top row are enlarged in the bottom row. (B) The number of VGLUT 1-positive synaptic puncta in autaptic neurons co-cultured with 5- (control), 9- and 16-week-old astrocytes (*n* = 33, 36 and 41 autaptic neurons, respectively). (C) Area (size) of the VGLUT 1-positive synaptic puncta, measured as the number of pixels. The data were obtained from the specimens used in (B).

The reduction in glutamate release could also be attributable to a decrease in a fourth factor, the number of synapses formed on the neurons. Therefore, we counted the number of glutamate-releasing (glutamatergic) synapses in the autaptic neuronal cultures (Figure 5). We used immunocytochemical staining for the glutamatergic nerve terminal marker VGLUT 1 (red puncta, Figure 5A), because the majority of the excitatory synapses in autaptic hippocampal neurons possess VGLUT 1 [38], and thus the number of VGLUT 1-staining puncta corresponds approximately to the number of excitatory synapses. The number of puncta labeled for VGLUT 1 was unaffected by the age of the astrocytes on which the neurons were cultured (control, 326.0±33.3; 9w, 322.0±37.2; 16w, 264.2±26.7, Figure 5B). There was also no change in size of the puncta (Figure 5C). These results suggest that synapse formation was not affected by astrocyte age.

During our morphological examination of nerve terminals, we noted a slight increase in the number of dendritic branches near soma in cultures with aged astrocytes. To evaluate this effect in greater detail, we identified the dendrites by immunocytochemical staining for the dendritic marker MAP2 (green, Figure 5A, lower panels), and quantitatively evaluated the pattern of dendritic branching by Sholl analysis (Figure S3) [32], [33]. We counted the number of dendritic branches that intersect concentric rings drawn at various radii from the soma to assess the number of dendritic branch points (Figure S3A). We found no difference in the total number of dendritic intersections (crossings) among the three groups (control, 114.55±6.73; 9w, 122.57±8.82; 16w, 101.41±7.06, Figure S3B). However, in the case of neurons co-cultured with aged astrocytes, crossings were maximal closer to the soma (control, 65.47±5.0 µm; 9w, 54.58±3.89 µm; 16w, 42.13±3.42 µm, Figure S3C). These results suggest that the dendritic branch points lie more proximally to the soma when astrocytes are aged, raising the possibility that neurons co-cultured with aged astrocytes experience a slightly retarded development. This is interesting in light of previous reports that neuronal dendrites branch at more distal sites as neuronal development progresses [49], [50], [51].

### Long-term Astrocyte Culture does not Change the Ratio of Postsynaptically Silent Synapses

The observed decrease in the amplitudes of evoked EPSCs in the presence of aged astrocytes (Figure 2) could be a consequence of conversion of active synapses to an inactive (silent) state. Silent synapses are present physiologically, and their proportion among the total plays an important role in neuronal development and synaptic plasticity [52], [53], [54]. The sites of silencing can be either post- or pre-synaptic, and these will be evaluated separately.

**Figure 6:**
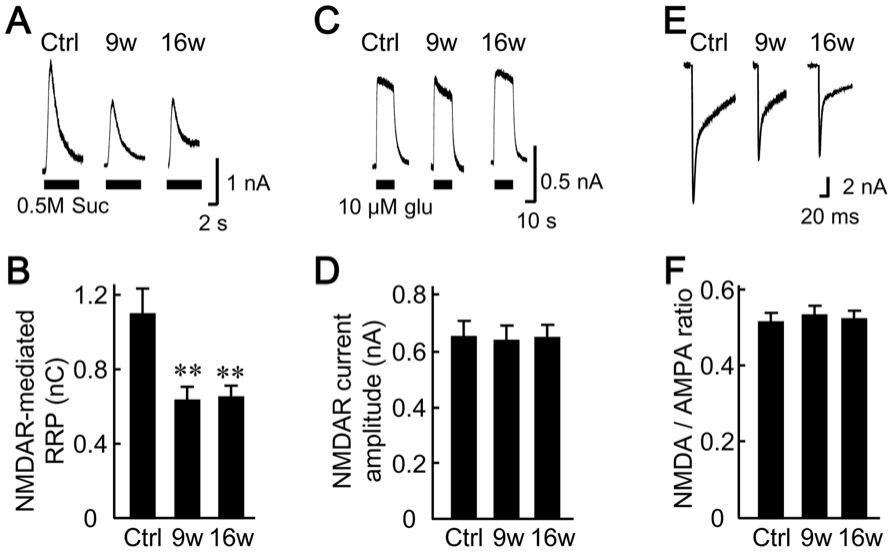
Astrocyte aging does not affect postsynaptically silent synapses. (A) Representative traces of responses to 0.5 M sucrose in autaptic neurons. RRP size was evaluated based on the responses to hypertonic sucrose solution as in Figure 4, but in this case, ionic currents through NMDA receptors (NMDAR) rather than AMPA receptors were measured. They were recorded at a Vh of +40 mV and in the presence of 10 µM glycine (maximizes the efficiency of NMDA receptor-mediated current recording), and of 10 µM CNQX (blocks the AMPA receptors). (B) Size of the RRP, measured as the electric charge carried by the NMDA receptors. Autaptic neurons were co-cultured with 5- (control), 9- and 16-week-old astrocytes (*n* = 82, 84, and 81 neurons, respectively). (C) Representative traces illustrating the responses of autaptic neurons to 10 µM glutamate for assessing the expression level of NMDA receptors. The experimental conditions were the same as in (A), except that glutamate rather than sucrose was used to induce ionic currents. (D) Amplitude of the NMDA receptor-mediated currents in autaptic neurons co-cultured with 5- (control), 9- and 16-week-old astrocytes (*n* = 64, 61, and 66 neurons, respectively). (E) Representative traces of evoked EPSCs. These were recorded from autaptic neurons, as in Figure 2 (Vh of −70 mV) except that the conditions favored the efficiency of NMDA receptor activity (i.e. extracellular solution contained 10 µM glycine and no Mg^2+^). Depolarization artifacts caused by the generated action currents have been removed for the sake of clarity. (F) Changes in the ratio of the NMDA and AMPA receptor expression levels in autaptic neurons co-cultured with 5- (control), 9- and 16-week-old astrocytes (*n* = 34, 35 and 38 neurons, respectively). The expression levels are represented by the amplitudes of evoked EPSCs mediated by NMDA and AMPA receptors. The NMDA and AMPA components of dual EPSCs were determined from current amplitudes measured 30–40 ms after the depolarizing stimulus was applied, and at the peak of the response. **, p<0.01.

Postsynaptic silencing results from the absence of postsynaptic AMPA receptors; NMDA receptors are present and glutamate is released normally from the presynaptic nerve terminals [55], [56], [57]. At non-silent synapses, the majority of synaptic transmission is carried out through AMPA receptors; the NMDA receptors are present at the same levels as in silent synapses and can bind the released glutamate, but usually remain non-permeable at the resting membrane potential due to a voltage-dependent block of the ion-conducting channel by extracellular Mg^2+^ [58]. Accordingly, postsynaptically silent synapses are classically defined as those that do not produce measurable ESPCs at the resting membrane potential (Mg^2+^ block of NMDA receptors and lack of AMPA receptors) but do so when the membrane is depolarized (unblocking of NMDA receptors) [55], [56]. Unfortunately, this method is not readily applicable to autaptic neurons, because continuous depolarization of an autaptic neuron via a patch pipette (e.g. +40 mV for Mg^2+^ unblock) will inactivate the voltage-dependent Na^+^ channels and therefore block the action potentials that would otherwise be generated. In the present study, the postsynaptically silent synapses were analyzed using the following three methods (Figure 6).

First, we replaced the action potential (step depolarization) for presynaptic activation and neurotransmitter release, by applying hypertonic sucrose solution and thereby forcing glutamate release from RRP vesicles (Figure 6A). We evaluated the NMDA receptors by removing the Mg^2+^ block at a depolarized Vh of +40 mV. This condition was combined with the application of an agonist of the glycine-binding site of NMDA receptors (10 µM glycine) and an antagonist of AMPA receptors (10 µM CNQX). Under these conditions, the NMDA receptor-mediated synaptic charge induced by the sucrose solution was decreased in the presence of aged astrocytes (control, 1.01±0.13 nC; 9w, 0.63±0.07 nC; 16w, 0.64±0.06 nC, Figure 6B). This effect on the RRP was consistent with that measured through the activated AMPA receptors at a Vh of −70 mV (Figure 4A and B), indicating that there was no age-dependent change in expression levels of the AMPA and NMDA receptors, and therefore in the number of non-silent synapses.

Second, we applied exogenous glutamate and measured the changes in the amplitude of the AMPA receptor-mediated current (determined by the expression level of AMPA receptors, Figure S2), and in the amplitude of the NMDA receptor-mediated current (determined by the expression level of NMDA receptors, Figure 6C and D). We had already shown that the AMPA receptor-mediated current induced by exogenous glutamate did not differ among the three groups of neurons grown on astrocytes of different ages (Figure S2B). We also did not observe an age-dependent change in the NMDA receptor-mediated current (control, 0.65±0.06 nA; 9w, 0.64±0.05 nA; 16w, 0.65±0.05 nA, Figure 6C and D) when recorded under the conditions described for Figure 6A. The lack of change in the glutamate-induced currents mediated by the AMPA and NMDA receptors indicates that the levels of expression of these receptors did not change.

Third, we assessed changes in ratios of the AMPA and NMDA receptors expressed at synapses (Figure 6E and F). Electrophysiologically, these values can be calculated from the relative amplitudes of the AMPA- and NMDA-receptor-mediated synaptic currents (relative expression levels) following an action potential (Figure 6E). NMDA receptors can be maximally activated by applying an extracellular solution that lacks Mg^2+^ and contains 10 µM glycine. Under these experimental conditions, the EPSC evoked by an action potential at a Vh of –70 mV has components with both fast and slow decays. The fast component is mediated by AMPA receptors, and the slow component by NMDA receptors [35], [59]. The presence of these dual EPSCs in individual neurons allows us to measure the amplitudes of the AMPA component at the peak of the EPSC, and the NMDA component at 30–40 ms after the peak. The ratio of NMDA to AMPA components did not differ among the three groups (control, 0.52±0.02; 9w, 0.55±0.02; 16w, 0.52±0.02, Figure 6F). Together, these findings suggest that the aged astrocytes did not affect the postsynaptically silent synapses.

### Long-term Astrocyte Culture does not Change the Ratio of Presynaptically Silent Synapses

**Figure 7:**
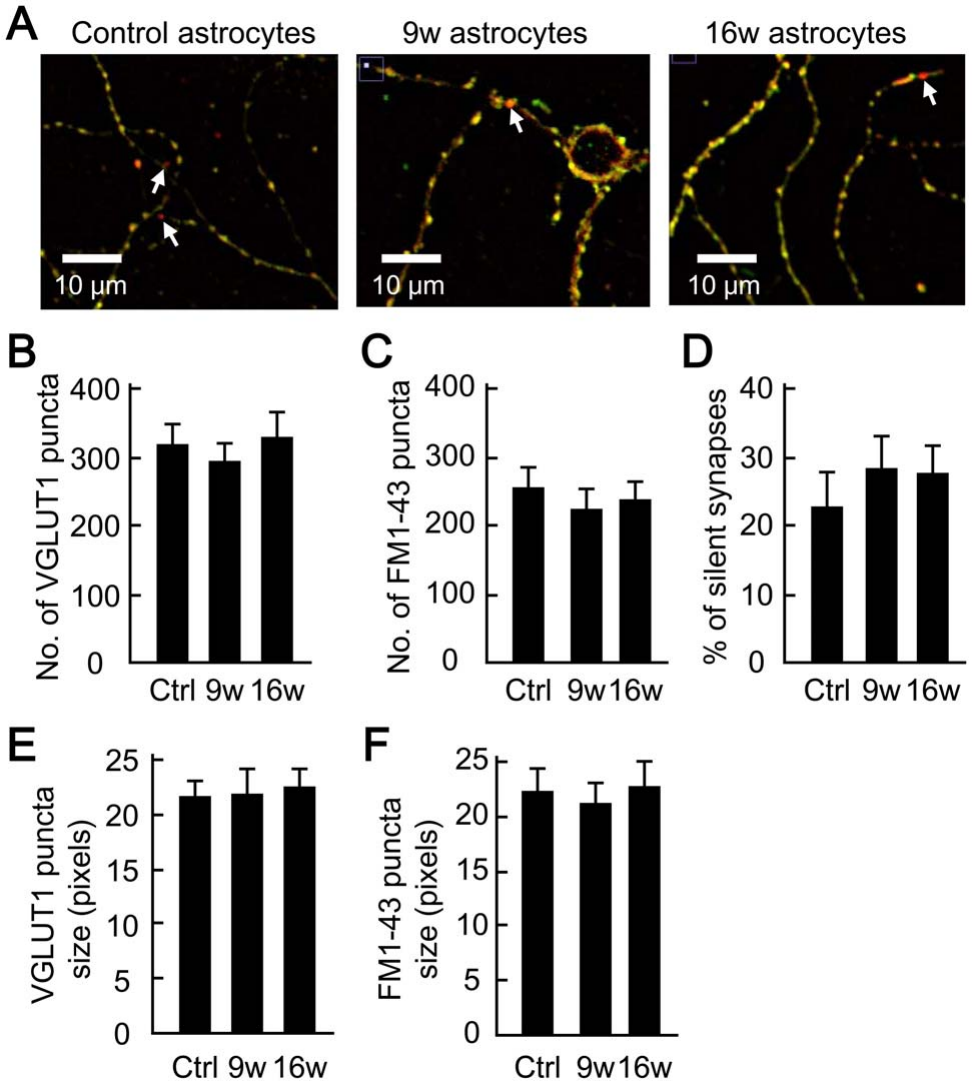
Astrocyte aging does not affect presynaptically silent synapses. (A) Representative images illustrating the functional (non-silent) synapses labeled with both 10 µM fixable FM1-43FX (green) and VGLUT 1 antibody (red), and the inactive (silent) synapses labeled with VGLUT 1 antibody only (arrows). Autaptic neurons were co-cultured with 5- (control), 9- and 16-week-old astrocytes. (B) The number of VGLUT 1-positive puncta in autaptic neurons co-cultured with 5- (control), 9- and 16-week-old astrocytes (*n* = 27, 20 and 22 neurons, respectively). (C) The number of FM1-43FX-positive puncta in autaptic neurons. Data in (C-F) were obtained from the same neurons used in (B). (D) Ratio of presynaptically silent synapses to total synapses, measured as the fraction of VGLUT 1-positive puncta that did not colocalize with FM1-43FX. (E) Area (size) of the VGLUT 1-positive puncta, measured as the number of pixels. (F) Area (size) of the FM1-43FX-positive puncta, measured as the number of pixels.

We next examined whether the ratio of presynaptically silent synapses is affected in the presence of aged astrocytes (Figure 7). Astrocyte-derived factors such as cholesterol and apolipoprotein E have been reported to play key roles in facilitating presynaptic release from synapses, and the synaptic maturation [6]. In previous studies, presynaptically silent synapses were identified as glutamatergic synapses that can be immunolabeled with the glutamatergic nerve-terminal marker VGLUT1 but do not possess recycling synaptic vesicles, and therefore are not labeled with the fluorescent marker of recycling vesicles, FM1-43, or its fixable form (FM1-43FX) [29], [30]. The styryl dye FM1-43 is loaded into endocytosed synaptic vesicles of non-silent (functional) nerve terminals [60].

The non-silent nerve terminals of the autaptic neurons were stained with FM1-43FX, by continuous depolarization (application of 45-mM KCl extracellular solution) in the presence of the dye (Figure S4, see Methods). After washout with dye-free extracellular solution, the stained nerve terminals were visible as fluorescent puncta (Figure S4A and B). To differentiate puncta from non-specific staining, we tested for dye unloading through exocytosis (Figure S4C). Time-lapse imaging showed that 2-min depolarization using 90-mM KCl solution led to a decrease in fluorescence intensity (destaining) at most of the fluorescent puncta (Figure S4C and D). This result indicates that nerve terminals with recycling vesicles can be stained and destained with FM1-43FX, and ascertains that FM1-43FX staining can be used for labeling non-silent synapses.

We identified the presynaptically silent synapses by combining the FM1-43FX labeling of non-silent synapses, with the retrospective VGLUT1 immuno-staining of all glutamatergic synapses (Figure 7) [29], [30]. Overlay of FM1-43FX (green, Figure 7A) and VGLUT1 (red) images demonstrated that the two signals are colocalized at many fluorescent puncta (yellow, non-silent synapses). In contrast, VGLUT1 signal in isolation was visible at a limited number of puncta (red, silent synapses, indicated by arrows in Figure 7A). The three groups exhibited no significant differences with respect to the number of VGLUT 1 puncta (control, 314.07±28.08; 9w, 288.58±25.53; 16w, 323.82±34.82, Figure 7B) or the number of FM puncta (control, 249.0±27.67; 9w, 216.95±28.50; 16w, 231.46±25.28, Figure 7C). As a consequence, the ratio of presynaptically silent synapses did not differ among the three groups of culture (control, 22.11±4.81%; 9w, 27.75±4.71%; 16w, 27.12±3.84%, Figure 7D). There was also no change in sizes of the VGLUT1 and FM puncta (Figure 7E and F). In our control cultures, ∼22% of total synapses were presynaptically silent, consistent with previously reported values [29], [37], [61], [62]. These data suggest that the presynaptically silent synapses were not affected by the long-term culture of astrocytes.

### Long-term Astrocyte Culture does not Affect the Expression of Active Zone Protein Bassoon

**Figure 8:**
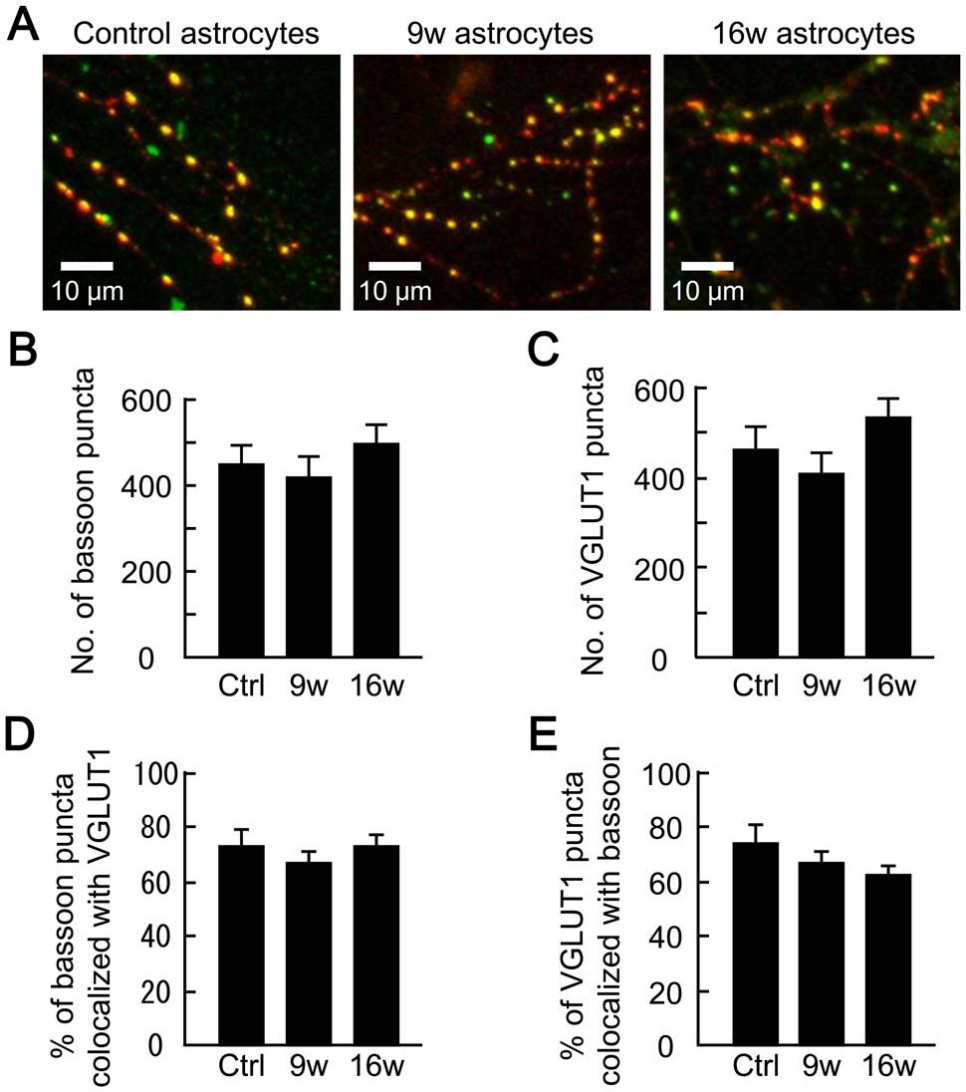
Astrocyte aging does not affect the colocalization of bassoon and VGLUT 1. (A) Representative images illustrating colocalization of the presynaptic proteins bassoon (green) and VGLUT 1 (red). Autaptic neurons were co-cultured with 5- (control), 9- and 16-week-old astrocytes. (B) The number of bassoon-positive puncta in autaptic neurons co-cultured with 5- (control), 9- and 16-week-old astrocytes (*n* = 30, 29 and 35 neurons, respectively). (C) The number of VGLUT 1-positive puncta in autaptic neurons. Data in (C-E) were obtained from the neurons analyzed in (B). (D) Percentage of bassoon-positive puncta that overlapped with VGLUT 1-positive puncta. Data were analyzed by assigning regions of interest in the bassoon images and transferring them to VGLUT 1 images of the same neurons. (E) Percentage of VGLUT 1 puncta that overlapped with bassoon puncta. Data were analyzed by assigning regions of interest in the VGLUT 1 images and transferring them to the bassoon images of the same neurons.

Our data indicate that the aged astrocytes would affect exocytotic features, namely the amplitude of evoked EPSCs, mEPSC frequency and RRP size (Figures 2, 3 and 4). We also found a lack of overt changes in endocytosis of the synaptic vesicles (number and size of stained FM puncta; Figure 7). These results prompted us to examine the structural integrity of nerve terminals involved in synaptic vesicle exocytosis. Exocytosis from the active zone of a nerve terminal is dependent on the orchestration of its structure and function by presynaptic proteins such as bassoon, piccolo, Munc13, RIM and CAST [37], [61], [63], [64], [65]. We evaluated the properties of bassoon as a representative (Figure 8), firstly because bassoon is 80–90% colocalized with numerous presynaptic and postsynaptic proteins in autaptic neuronal cultures [61], and secondly because our electrophysiological data are very similar to those for bassoon-deficient synapses [61]. Immunocytochemical analysis revealed (Figure 8A) that there was no change in the numbers of bassoon-positive puncta (control, 444.47±44.54; 9w, 415.31±43.81%; 16w, 491.26±42.11, Figure 8B) or VGLUT 1-positive puncta (control, 458.67±44.36; 9w, 400.97±43.71; 16w, 527.09±36.79, Figure 8C) in our samples. Bassoon and VGLUT-1 were high colocalized (∼65%; Figure 8D and E), in all the three groups. These data indicate that bassoon-dependent morphological features are not affected by astrocyte aging.

### Developmental Changes in Synaptic Transmission

**Figure 9:**
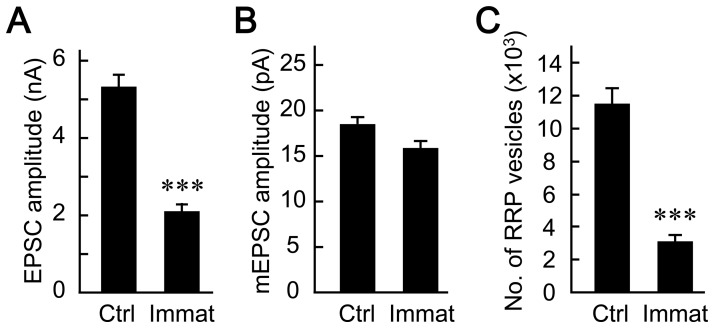
Synaptic transmission changes developmentally. (A) Amplitudes of evoked EPSCs recorded from mature (control, 13–15 DIV, *n* = 123 neurons) and immature autaptic neurons (7–9 DIV *n* = 102 neurons). Both types of neurons were co-cultured with 4- to 5-week-old astrocytes. (B) Amplitudes of mEPSCs (means of mean amplitudes) recorded from individual mature (*n* = 54 neurons) and immature autaptic neurons (*n* = 56 neurons). (C) The numbers of synaptic vesicles in the RRPs of mature (*n* = 54) and immature (*n* = 56) autaptic neurons. Data were obtained from the same neurons analyzed in (B). RRP data were analyzed as in Figure 4B. ***, p<0.001.

The reduction of synaptic transmission in the presence of aged astrocytes is reminiscent of observations from immature neurons in the hippocampal mass culture [66]. In order to assess developmental change of this type in autaptic cultures, we maintained the hippocampal neurons on microislands for 7 to 9 days (immature) and compared their synaptic properties with those of counterparts cultured for 13–15 days (control) (Figure 9). In both groups, the ages of the astrocytes comprising the microislands were kept constant at 4–5 weeks. The amplitudes of the evoked EPSCs were smaller in immature neurons than in control neurons (control, 5.66±0.33 nA; immature, 2.37±0.21 nA, Figure 9A), whereas the amplitudes of mEPSCs were not different from those of controls (control, 19.70±0.93 pA; immature, 16.8±0.69 pA, Figure 9B), suggesting that a presynaptic change occurs during development. The number of synaptic vesicles in the RRP was estimated by applying hypertonic sucrose, and was found to be significantly smaller in immature neurons than in control neurons (control, 11262±851 vesicles; immature, 2673±303 vesicles, Figure 9C). These synaptic properties of immature neurons were identical to those of control neurons co-cultured on aged astrocytes (Figures 2, 3 and 4). These results indicate that aged astrocytes may delay the developmental or maturation of neurons.

## Discussion

Astrocytes are key players in not only classical processes such as the formation, maturation and elimination of synapses, but also in the modulation of synaptic transmission among vertebrate neurons [1], [2], [3], [4], [5], [67]. The diverse outcomes in the studies demonstrating these points show that astrocytes and synapses are highly interdependent. Collectively these studies established the concept of the “tripartite synapse”, which is composed of not only presynaptic and postsynaptic neuronal components, but also synaptically associated glial cells [68], [69].

To our knowledge, our work is the first to extensively analyze the influence of astrocyte age on synaptic transmission. We have found that astrocytic aging is accompanied by reductions in the size of the RRP (Figure 4B), the magnitude of action-potential-induced glutamate release (i.e., the EPSC amplitude, Figure 2), and the frequency of miniature glutamate release events (mEPSC frequency, Figure 3B). We posit that among these presynaptic features, suppression of RRP size is the fundamental action, since this parameter is a determinant of both EPSC amplitude [which is expressed as: (EPSC amplitude) = (RRP size)×(vesicular release probability)] and mEPSC frequency [40], [70], [71]. This functional effect was consistently observed in 9- and 16-week-old astrocyte cultures, which also showed morphological features of senescence (Figure 1). The reduction in RRP size was a specific effect, not simply due to detrimental effects of astrocyte aging, because most critical neuronal features were retained irrespective of astrocytic age. Such features include: mEPSC amplitude (Figure 3C), vesicular release probability (Figure 4C), paired-pulse ratio (Figure 4D), Ca^2+^ sensitivity of vesicular release (Figure 4C and D), number and size of glutamatergic nerve terminals (Figure 5), expression of postsynaptic NMDA and AMPA receptors (Figures 6 and S2), ratios of post- or pre-synaptically silent glutamatergic synapses (Figures 6F and 7), expression of VGLUT1 (Figures 5, 7 and 8) and bassoon (Figure 8) at the presynaptic terminals, neuronal membrane excitability (Figure S1), and the total extent of dendritic arborization (Figure S3B).

### Aged Astrocytes Promote Presynaptically Immature Synapses

Hippocampal neurons in culture develop rapidly during the first and second weeks after plating (Figure 9), as suggested previously [66], [72]. Based on the developmental features of immature neurons (7–9 DIV, Figure 9) [66], which closely resemble those of more mature neurons cultured on aged astrocytes (13–15 DIV, Figures 2, 3 and 4), we postulate that the phenotype observed in neurons in the latter scenario was caused by an increase in the number of presynaptically immature synapses. This notion is partially consistent with our data showing a proximal location of dendritic branches (Figure S3C), as dendritic extensions in the CNS develop during neuronal maturation [49], [50], [51]. Thus our data indicate that aged astrocytes promote presynaptically immature synapses by retarding synapse maturation, resulting in a smaller size of readily releasable pool of synaptic vesicles.

Alternatively, it is theoretically possible that the aging astrocytes in our study converted previously active synapses into immature synapses by increasing the ratio of silent synapses. However, our data fail to show a correlation between astrocyte aging and the fraction of silent synapses, either postsynaptically (Figures 6 and S2) or presynaptically (Figure 7). We therefore rule out an involvement of silent synapses in the effects of aged astrocytes.

### Implications for Physiology and Pathophysiology

In certain parts of the brain including the hippocampus, synaptogenesis and astrogenesis occur throughout the entire lifespan [73]. This raises the possibility that the tripartite synapse is under dynamic control of neurons and astrocytes of different ages. Indeed, an age-dependent effect was clearly demonstrated in the mammalian cerebral cortex. The presence of immature cortical astrocytes is physiologically required for visual plasticity, and the critical period ends with the maturation of astrocytes [74], [75]. In contrast to the knowledge of the effects of immature astrocytes on synaptic transmission, those of aged astrocytes have remained largely elusive. A reduction in glutamate release during aging appears to occur in several brain regions, even when a suppressed glutamate uptake from the extracellular space is taken into consideration [76]. However, the measurement of glutamate release at the whole-tissue level can be compromised by potential changes in other factors, such as the neuronal excitability, number of neurons, glial release of glutamate [76], and number of synapses. The reduced RRP size found in the current study at the single-neuron level might be partly responsible for the reduction in glutamate release.

Our finding suggests that aged astrocytes lack a capacity to promote full presynaptic development, which also supports the notion that, in addition to regulating synapses under physiological conditions, astrocytes can contribute to synaptic abnormalities under pathological conditions. For example, in a neuron-astrocyte co-culture system, astrocytes isolated from a mouse model of fragile X syndrome produced delays in the branching and lengthening of dendrites of wild-type neurons [77], [78]. Furthermore, astrocytes induce severe neuronal abnormalities in other neurodevelopmental diseases, such as the Rett syndrome (caused by a deficiency for methyl-CpG-binding protein 2, MeCP2) [79], [80] and Alexander’s disease (caused by a mutated GFAP; for review, see [81]). Currently, in any disease models, it is not yet fully understood how synaptic transmission is manipulated by pathophysiological abnormalities of astrocytes. The results reported here, in conjunction with the above-mentioned recent results implicating astrocyte defects in diseases, indicate the importance of further elucidating the pathophysiological roles of astrocytes in regulating the synaptic transmission.

In the present study, the effects of astrocyte aging were clarified using a simplified *in vitro* system. The co-culture of neurons with astrocytes of different ages, in combination with the suppression of astrogenesis by antimitotic agents, was aimed at controlling the time course of aging. Notwithstanding its usefulness to this point, this system has at least two features that require improvement. One is the loss of cell-cell interaction cues when astrocytes are maintained for a prolonged period in the absence of other cell types, such as neurons or endothelial cells. Aging in this context might unnaturally eliminate the contributions of other cells to astrocytic maturation and senescence. The second is the suppression of astrogenesis, which occurs during the early postnatal period [82] and even in the adult [83], [84]. Other *in vitro* systems have seen improvements through, e.g. the co-culture of purified retinal ganglion cells with astrocytes [2], [4], [10], acute isolation of aged astrocytes [12] and culturing of induced pluripotent stem cells obtained from aged animals [85], and some of these approaches could theoretically be applied to our system. Furthermore, experiments using *in vitro* systems in combination with *in vivo* systems will be useful in examining the complex interplay between astrocytes, synapses and other cells.

## Methods

### Ethics Statement

All procedures regarding animal care were performed in strict accordance with the rules of the Experimental Animal Care and Welfare Committee of Fukuoka University (comparable to NIH guidelines), following approval of the experimental protocol by this body (Permit Numbers: 1002364 and 1203540). Cells for primary culture were obtained from newborn mice after decapitation under ether anesthesia, and every effort was made to minimize suffering.

### Mass and Microisland Cultures of Astrocytes

Microisland cultures of cerebral cortical astrocytes were prepared as reported previously [25], [26], [27]. Astrocytes were obtained from cerebral cortices of newborn (P0) ICR mice (Kyudo, Japan). Cerebral cortices were removed from brains in cold Hank’s Balanced Saline Solution (HBSS, Invitrogen) and dissociated with 0.05% trypsin-EDTA (Wako, Japan). The cells were then plated in the plating medium composed of Dulbecco’s Modified Eagle Medium with GlutaMAX™ and pyruvate (DMEM, Invitrogen), supplemented with 10% fetal bovine serum (FBS, Invitrogen) and 0.1% MITO+ Serum Extender (BD Biosciences), in 75 cm^2^ culture flask (250 ml, BD falcon). The next day, the culture flask was gently rinsed once with the plating medium to remove non-adherent cells. When the culture reached confluence 2 weeks later, microglia and other small cells were removed by tapping the culture flask several times. The medium was replaced by a fresh plating medium, at which point the antimitotic agents 5-fluoro-2′-deoxyuridine (8 µM, Sigma-Aldrich) and uridine (20 µM, Sigma-Aldrich) were added to curtail glial proliferation and maintain culture purity. Some culture flasks were incubated for additional 4 or 11 weeks, with the plating medium containing the antimitotic agents exchanged every week. The cultured astrocytes showed progressive aging in the presence of antimitotic agents (see Figure 1).

For the mass cultures of astrocytes, sterilized 22-mm coverslips (thickness No. 1, Matsunami, Japan) were treated with a 1∶1 mixture of rat-tail collagen (final concentration of 1 mg/ml, BD Biosciences) and poly-D-lysine (final concentration of 0.5 mg/ml, Sigma-Aldrich), by uniform spreading with a cotton swab.

For the culturing of astrocytes as microislands (in the absence of neurons), the sterilized 22-mm coverslips were pre-coated uniformly with 0.5% liquefied agarose to prevent cells from attaching. The next day, a mixture of collagen and poly-D-lysine was applied on top of the agarose layer, using a custom-made dot-stamp designed to deposit substrate in 300-µm squares with 400-µm edge-to-edge intervals.

Adherent cells in the culture flasks, cultured for 2, 6 and 13 weeks, were trypsinized and plated on either the coverslips prepared for mass culture, at a density of 26,000 cells/cm^2^, or on the dot-stamped coverslips prepared for microisland culture, at a density of 6,000 cells/cm^2^. In both cases, the cells were cultured in plating medium without antimitotic agents. When the astrocytes formed a monolayer (within a week), antimitotic agents were added to the plating medium. Two weeks before the experiments were initiated, the conditioned media in the mass cultures (Figure 1A, B and C) and microisland cultures (Figure 1D, E and F) were replaced with Neurobasal-A medium (Invitrogen) containing 2% B27 supplement (Invitrogen), in a manner identical to that for the autaptic neuronal cultures. Although antimitotic agents were not present in the serum-free Neurobasal-A medium, glial proliferation was inhibited under these conditions (data not shown).

### ß-Galactosidase Staining

Astrocytes mass-cultured on coverslips were washed twice with phosphate-buffered saline (PBS) and fixed in PBS containing 4% paraformaldehyde (PFA) for 20 min at room temperature. Senescence in astrocytic cultures was assessed using ß-galactosidase-based Senescence Cells Staining Kit, according to the manufacturer’s instructions (CS0030-1KT, Sigma-Aldrich). Astrocytes stained blue at pH 6 (4 mM potassium ferricyanide solution and 4 mM potassium ferrocyanide solution) were visualized using a light microscope in bright-field mode. The astrocyte nuclei were visualized by counterstaining with DAPI contained in the mounting medium (ProLong® Gold antifade mounting reagent, Invitrogen). The numbers of positively stained cells in 3x3 mm^2^ square areas were counted, three times independently per culture batch, and were averaged. Data were obtained from three different cultures.

### Autaptic Neuron Culture

Hippocampi (CA3-CA1 regions) were isolated from the brains of newborn ICR mice different from the one used to prepare astrocytic cultures, and enzymatically dissociated in DMEM containing papain (2 units/ml, Worthington), for 60 min at 37°C. The cells were plated at a density of 1,500 cells/cm^2^, onto the astrocyte microislands. Before the dissociated hippocampal neurons were plated, the conditioned medium of the astrocyte microisland culture was replaced with Neurobasal-A medium containing 2% B27 supplement. For evaluating the synaptic parameters in autaptic neurons (Figures 2, 3, 4, 5, 6, 7, 8 and Figures S1, S2, S3, S4), hippocampal neurons were then co-cultured for 13–16 days on microisland astrocytes that had already been cultured for different periods (3, 7 and 14 weeks). For evaluating the synaptic transmission in immature autaptic neurons (Figure 9), the neurons were co-cultured for 7–9 days on microisland astrocytes that had already been cultured for 3 weeks.

### Electrophysiology of Autaptic Neuronal Cultures

All electrophysiological experiments were performed using autaptic neuronal cultures [25], [26], [34], [35], [36], [37], [38], [39]. Recordings were performed on neuronal 13–16 days *in vitro* (DIV), to ensure that synaptic responses were stable with reliable space clamping. Synaptic responses were recorded using a patch-clamp amplifier (MultiClamp 700B, Molecular Devices), in the whole-cell configuration under the voltage-clamp mode, at a holding potential (Vh) of –70 mV unless otherwise specified, and at room temperature in all cases. Patch-pipette resistance was 4–5 MΩ, and 70–90% of access resistance was compensated. Autaptic neurons showed synaptic transmission in response to an action potential elicited by a brief (2 ms) somatic depolarization pulse (to +0 mV) from the patch pipette. The synaptic responses were recorded at a sampling rate of 20 kHz, and were filtered at 10 kHz. Data were excluded from analysis if a leak current >300 pA was observed. Data were analyzed off-line using Axograph X 1.2 software (AxoGraph Scientific). The mEPSCs were detected with an amplitude threshold of 5 pA. All the traces were visually examined to protect against software errors.

RRP size (i.e., the number of synaptic vesicles in the RRP) was calculated in the following way:

1. The total charge of the RRP was measured, as the transient component of the electric charge of an excitatory postsynaptic current (EPSC) elicited by a 10-s application of 0.5-M sucrose. This measurement is based on the fact that sucrose forces the release of all synaptic vesicles in the RRP [40]. The transient component of the sucrose-induced EPSCs estimates the total charge of the RRP, whereas the steady-state component estimates the charge of the non-primed (reserve) pool, which is mobilized to the RRP as the original RRP is depleted [25].
2. An electric charge of a single synaptic vesicle was measured from the same neuron, as the averaged electric charges of individual miniature excitatory postsynaptic currents (mEPSCs). mEPSCs were recorded in the presence of the Na^+^ channel blocker tetrodotoxin (TTX, 0.5 µM), and spontaneously (i.e. in the absence of any stimuli).
3. The size of the RRP was calculated by dividing the charge of the sucrose-induced transient EPSC by the charge of the mEPSC; i.e. RRP size = (charge of RRP)/(charge of mEPSC).

Vesicular release probability (P_vr_) was defined as the probability of release of individual synaptic vesicles in response to an action potential, and was calculated as follows:

1. The electric charge of an EPSC evoked by a single action potential was measured. This represents the fraction of RRP vesicles released by an action potential.
2. The total charge of the RRP was measured from the same neuron, as described above.
3. The P_vr_ was calculated by dividing the EPSC charge by the sucrose-induced transient EPSC; i.e. P_vr_ = (charge of action potential-induced EPSC)/(charge of RRP).

### Immunocytochemistry

Autaptic hippocampal neurons were fixed in PBS containing 4% PFA for 20 min at room temperature, and then blocked and permeabilized with PBS containing 5% normal goat serum and 0.1% Triton X-100, for 30 min. After blocking, the samples were incubated overnight at 4°C with the following primary antibodies: anti-microtubule-associated protein 2 (MAP 2, guinea-pig polyclonal, antiserum, Synaptic Systems, 1∶1000 dilution), anti-vesicular glutamate transporter 1 (VGLUT 1, rabbit polyclonal, affinity purified, Synaptic Systems, 1∶2000 dilution), anti-bassoon (guinea-pig polyclonal, Synaptic Systems, 1∶2000 dilution) or anti-glial fibrillary acidic protein (GFAP, rabbit polyclonal, Synaptic Systems, 1∶1000 dilution) in a humidity chamber. Autaptic neurons were incubated with appropriate species-specific fluorochrome-conjugated goat secondary antibodies (Alexa Fluor 488 for MAP 2 or bassoon, and Alexa Fluor 594 for VGLUT 1 or GFAP, 1∶400 dilutions, Invitrogen) for 1 hr at room temperature. Double immunocytochemical staining was performed using a combination of MAP 2 and VGLUT 1, or a combination of bassoon and VGLUT 1. Astrocyte nuclei were visualized by counterstaining with DAPI contained in the mounting medium (ProLong® Gold antifade mounting reagent, Invitrogen).

### Imaging of Active Synapses using FM1-43FX and Immunocytochemistry

Functionally active synapses were quantified in autaptic neuronal cultures, using fixable *N*-(3-triethylammoniumpropyl)-4-(4-(dibutylamino)styryl) pyridinium dibromide (FM1-43FX, Invitrogen) [28]. The recycling synaptic vesicles were loaded with 10 µM FM1-43FX, with application of an extracellular solution containing 45 mM KCl for continuous depolarization, and the NMDA receptor antagonist (2R)-amino-5-phosphonovaleric acid (APV, 50 µM, Sigma-Aldrich) and the AMPA receptor antagonist 6-cyano-7-nitroquinoxaline-2,3-dione (CNQX, 10 µM, Sigma-Aldrich), for 2 min at room temperature. The samples were rinsed with an extracellular solution containing 500 µM Advasep-7 (Sigma-Aldrich) for 5 s, to eliminate non-specific dye labeling [29], [30], and washed with the dye-free extracellular solution for 10 min. The specificity of FM1-43FX labeling at active terminals was confirmed by depolarizing the autaptic neurons using the extracellular solution containing 90 mM KCl, 50 µM APV and 10 µM CNQX, for 2 min, and observing the loss of fluorescence. The high-K^+^ extracellular solutions were prepared by equimolar substitution of KCl for NaCl. The autaptic neurons were imaged using an inverted microscope (Eclipse-TiE, Nikon, Japan) equipped with an EMCCD camera (Cascade 512F, Roper Scientific, USA). FM1-43FX was excited using a 480-nm LED (Rapp OptoElectronic, Germany) at 50% intensity, and imaged with an objective lens (S Plan Fluor, 40x, NA 0.6, Nikon, Japan). Sixteen-bit images were acquired at 1 frame/s, 300-ms exposure time, EM gain of 20, and without binning, using the PM capture Pro software (Photometrics, USA). Spatial resolution at the specimen level was 0.2 µm×0.2 µm/pixel.

The ratio of inactive (presynaptically silent) synapses was estimated by carrying out VGLUT 1 staining after FM1-43FX labeling, generally in accordance with previous reports [29], [30] but with some modifications. Briefly, after FM1-43FX labeling, the autaptic hippocampal neurons were fixed in 4% PFA and 0.2% glutaraldehyde in PBS for 20 min at room temperature. They were then blocked and permeabilized with PBS containing 5% normal goat serum and 0.02% Triton X-100 for 20 min. This low detergent concentration was chosen because FM1-43FX is very sensitive to it [29], [30]. The use of 0.02% allowed for permeabilization of the cell membrane without removal of FM dye from the positive puncta in autaptic neurons, whereas the use of 0.04% removed the FM dye. Next, the autaptic neurons were incubated with anti-VGLUT1 antibody for 3 hrs at room temperature, washed, and incubated with Alexa Fluor 594-conjugated secondary antibody for 30 min at room temperature.

### Image Acquisition and Quantification

Confocal images of autaptic neurons were obtained using an objective lens (C-Apochromat, 40×, NA 1.2, Carl Zeiss, Germany), with sequential acquisition settings at the high-resolution (1024×1024 pixels) of the confocal microscope (LMS710, Carl Zeiss, Germany). The parameters of each image were optimized for the z-stack setting (a depth of 9 µm with 0.5 µm steps) and a pinhole setting (1 Airy unit). The depth range sufficiently covered the thickness of the neurites in our autaptic culture. The confocal microscope settings were kept the same for all scans in each experiment. Single autaptic neurons were selected for analysis in a blinded fashion, based on MAP 2 fluorescence or differential interference contrast (DIC) images. For double-imaging of synaptic puncta, fluorescent images were acquired sequentially from the channel visualizing VGLUT 1 (red emission) and the channel visualizing the second marker (green emission).

The sizes and numbers of synaptic puncta were detected and verified visually using the ImageJ software (1.46j, Wayne Rasband, NIH). The procedures used to analyze synaptic puncta were published previously [31]. A punctum was selected with a size threshold of larger than or equal to 5 pixels. There is a possibility that glial VGLUT1 was included in the analysis, although it would contribute little within the astrocyte microisland. The absolute intensity of fluorescence puncta was corrected by subtracting the background intensity measured from a bare area of the coverslip. In the case of FM staining, some areas of fluorescence could not be resolved owing to a high density of synapses, and therefore the number of synaptic puncta was estimated by dividing the total fluorescent area by the mean size of FM1-43-positive puncta in those regions where each punctum was clearly identified. The number of presynaptically silent synapses was estimated by identifying the regions of interest in the VGLUT 1 image and transferring them to the FM image, and then quantifying those that failed to colocalize with FM signal. A similar approach was used to estimate the colocalization of VGLUT 1 and bassoon.

Differences in dendritic branching patterns were quantified using the Sholl analysis [32], [33], which is accessible as a Plug-in (v1.0, The Ghosh lab) for ImageJ. Briefly, concentric circles were drawn in 7.5-µm-radius steps, with the center placed on the soma. The radial distance from the center of the soma was measured when a dendrite crossed any of the concentric circles.

### Solutions

The standard extracellular solution for the patch-clamp experiments was (in mM) NaCl 140, KCl 2.4, HEPES 10, glucose 10, CaCl_2_ 2, MgCl_2_ 1, pH 7.4, with an adjusted osmotic pressure of 315–320 mOsm. Patch pipettes were filled with an intracellular solution composed of (in mM) K-gluconate 146.3, MgCl_2_ 0.6, ATP-Na_2_ 4, GTP-Na_2_ 0.3, creatine phosphokinase 50 U/ml, phosphocreatine 12, EGTA 1, HEPES 17.8, pH 7.4. Hypertonic solutions for determining the RRP size were prepared by adding 0.5 M sucrose to the standard extracellular solution. The extracellular solutions were applied using a fast-flow application system (SF-77B, Warner Instruments). Each flow pipe has a large diameter (430 µm), ensuring that the solution is applied to all parts of an autaptic neuron on an astrocytic microisland (300×300 µm). This configuration is necessary if the application of sucrose is to induce the release of RRP vesicles from all nerve terminals of the recorded neuron. All chemicals were purchased from Sigma-Aldrich, except where otherwise specified.

### Statistical Analysis

Data are expressed as means ± s.e.m. Statistical analyses were performed using one-way analysis of variance (ANOVA) with a post hoc Tukey-HSD test for the comparison of three groups. Differences were considered to be significant when *p*<0.05 vs. all data from the control case, i.e. neurons co-cultured with astrocytes that had been cultured for 5 weeks.

## Supporting Information

Figure S1 **Astrocyte aging does not lead to changes in the Na^+^ and K^+^ currents responsible for the generation of action potentials.** (A) Representative autaptic currents elicited by a 2-ms step-depolarization from −70 to 0 mV. The negative and positive deflections represent the voltage-dependent Na^+^ and K^+^ currents, respectively, that underlie the action potentials [46]. (B) Amplitudes of the Na^+^ currents recorded from autaptic neurons co-cultured with 5- (control), 9- and 16-week-old astrocytes (*n* = 67, 64 and 70 neurons, respectively). (C) Amplitudes of the K^+^ currents recorded from the autaptic neurons analyzed in (B).
(TIF)

Figure S2 **Astrocyte aging does not affect the expression level of postsynaptic AMPA receptors.** (A) Representative responses of autaptic neurons to 10 µM glutamate in the extracellular solution. The Vh was −70 mV, and NMDA receptors were completely blocked by the continuous presence of an NMDA receptor blocker (50 µM APV). (B) Amplitudes of the glutamate-induced currents mediated by AMPA receptors (AMPARs). The autaptic neurons were co-cultured with 5- (control), 9- and 16-week-old astrocytes (*n* = 32, 32, and 29 neurons, respectively).
(TIF)

Figure S3 **In the presence of aged astrocytes, dendritic branch points lie closer to the somata.** (A) Sholl analysis demonstrating the number of MAP2-positive dendrites that cross concentric rings drawn at the indicated distances from the soma. Autaptic neurons were co-cultured with 5- (control, black), 9- (gray), and 16-week-old astrocytes (red) (*n* = 33, 36, and 41 neurons, respectively). (B) Total number of crossings. (C) Peak point of dendritic crossings, i.e. the distance at which the number of crossings was the highest in individual autaptic neurons. *, p<0.05; ***, p<0.001.
(TIF)

Figure S4 **Stained FM puncta represent functional (non-silent) nerve terminals that can release the dye on stimulation.** (A) Representative images of hippocampal autaptic neuron, observed using phase-contrast optics. Astrocytes were 5-weeks old at the time of imaging. Square box in the left panel is enlarged in the right panel. (B) Fluorescence images of the boxed region (A) showing FM1-43FX staining. After continuous depolarization (1-min, high-K^+^ stimulation), the FM intensity was decreased (destained), confirming that the puncta stained with FM1-43FX were functional nerve terminals. (C) FM intensity over time, as visualized by time-lapse imaging. Each trace represents the averaged kinetics of 30–40 fluorescent puncta in a single neuron. Data were normalized for the average intensity during a 1-min period before stimulation. The fluorescent puncta were selected using a procedure described in a previous report [28]. (D) Absolute intensity of FM1-43FX puncta before and after stimulation with high-K^+^ solution (*n* = 6 neurons). Absolute intensities were measured during 1-min periods before and after stimulation. **, p<0.01.
(TIF)
