# Correction: Long-Term Culture of Astrocytes Attenuates the Readily Releasable Pool of Synaptic Vesicles

**DOI:** 10.1371/annotation/9dd1f25a-55e9-4968-9f70-929d1b8d5064

**Published:** 2012-11-08

**Authors:** Hiroyuki Kawano, Shutaro Katsurabayashi, Yasuhiro Kakazu, Yuta Yamashita, Natsuko Kubo, Masafumi Kubo, Hideto Okuda, Kotaro Takasaki, Kaori Kubota, Kenichi Mishima, Michihiro Fujiwara, N. Charles Harata, Katsunori Iwasaki

There were errors in the Funding section. The correct funding information is as follows: This work was supported by Grant-in-Aid for Young Scientists (B) 23700476 from the Japan Society for the Promotion of Science, a grant from Naito Foundation and the Suzuken Memorial Foundation to SK, and grants from Dystonia Medical Research Foundation, Edward Mallinckrodt, Jr. Foundation, National Science Foundation and Whitehall Foundation to NCH. The funders had no role in study design, data collection and analysis, decision to publish, or preparation of the manuscript. No additional external funding was received for this study. 

