# Supplementary figures and images for: Long-Term Culture of Astrocytes Attenuates the Readily Releasable Pool of Synaptic Vesicles
Source: PLoS One. 2012 Oct 26;7(10):e48034. doi: 10.1371/journal.pone.0048034 (PMC3482238; doi:10.1371/journal.pone.0048034)

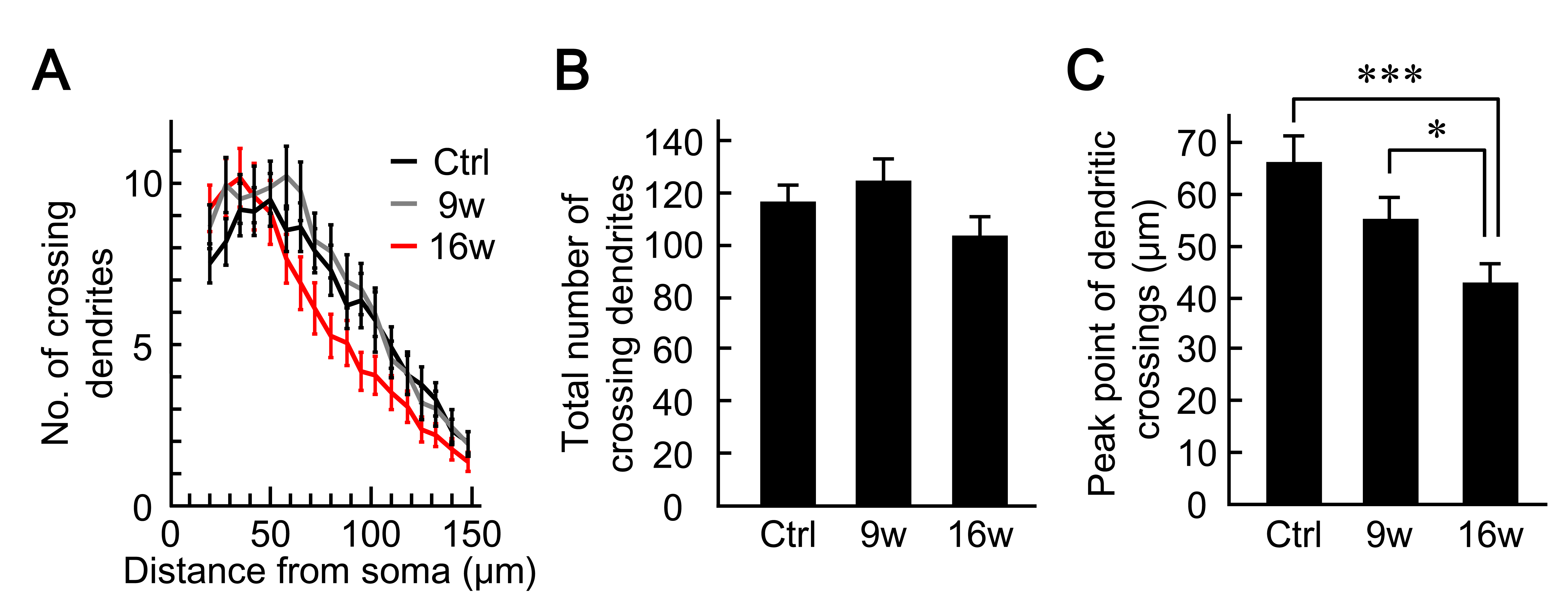

Supplement: Figure S3 — In the presence of aged astrocytes, dendritic branch points lie closer to the somata. (A) Sholl analysis demonstrating the number of MAP2-positive dendrites that cross concentric rings drawn at the indicated distances from the soma. Autaptic neurons were co-cultured with 5- (control, black), 9- (gray), and 16-week-old astrocytes (red) (n = 33, 36, and 41 neurons, respectively). (B) Total number of crossings. (C) Peak point of dendritic crossings, i.e. the distance at which the number of crossings was the highest in individual autaptic neurons. *, p<0.05; ***, p<0.001. (TIF) [file pone.0048034.s003.tif]

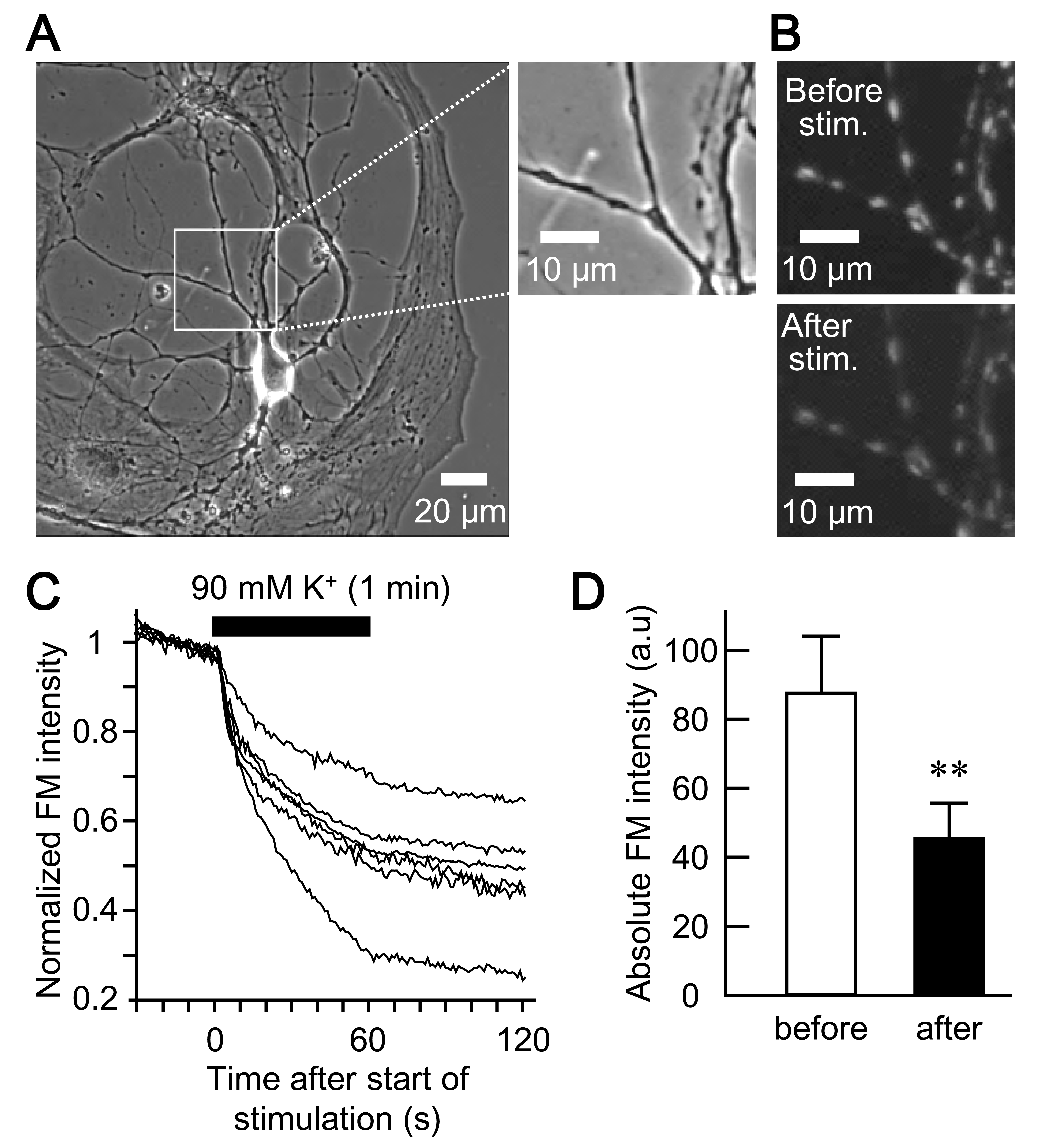

Supplement: Figure S4 — Stained FM puncta represent functional (non-silent) nerve terminals that can release the dye on stimulation. (A) Representative images of hippocampal autaptic neuron, observed using phase-contrast optics. Astrocytes were 5-weeks old at the time of imaging. Square box in the left panel is enlarged in the right panel. (B) Fluorescence images of the boxed region (A) showing FM1-43FX staining. After continuous depolarization (1-min, high-K+ stimulation), the FM intensity was decreased (destained), confirming that the puncta stained with FM1-43FX were functional nerve terminals. (C) FM intensity over time, as visualized by time-lapse imaging. Each trace represents the averaged kinetics of 30–40 fluorescent puncta in a single neuron. Data were normalized for the average intensity during a 1-min period before stimulation. The fluorescent puncta were selected using a procedure described in a previous report [28]. (D) Absolute intensity of FM1-43FX puncta before and after stimulation with high-K+ solution (n = 6 neurons). Absolute intensities were measured during 1-min periods before and after stimulation. **, p<0.01. (TIF) [file pone.0048034.s004.tif]
